# The mechanism of m^6^A methyltransferase METTL3-mediated autophagy in reversing gefitinib resistance in NSCLC cells by β-elemene

**DOI:** 10.1038/s41419-020-03148-8

**Published:** 2020-11-11

**Authors:** Shuiping Liu, Qiujie Li, Guohua Li, Qin Zhang, Lvjia Zhuo, Xuemeng Han, Mingming Zhang, Xiaying Chen, Ting Pan, Lili Yan, Ting Jin, Jianjun Wang, Qun Lv, Xinbing Sui, Tian Xie

**Affiliations:** 1 College of Pharmacy, School of Medicine, Hangzhou Normal University, Hangzhou, Zhejiang 311121 China; 2 Key Laboratory of Elemene Class Anti-Cancer Chinese Medicines; Engineering Laboratory of Development and Application of Traditional Chinese Medicines; Collaborative Innovation Center of Traditional Chinese Medicines of Zhejiang Province, Hangzhou Normal University, Hangzhou, Zhejiang 311121 China; 3 Department of Respiratory medicine, the Affiliated Hospital of Hangzhou Normal University, School of Medicine, Hangzhou Normal University, Hangzhou, Zhejiang 310015 China

**Keywords:** Cancer epigenetics, Lung cancer

## Abstract

N^6^-methyladenosine (m^6^A) modification can alter gene expression by regulating RNA splicing, stability, translocation, and translation. Emerging evidence shows that m^6^A modification plays an important role in cancer development and progression, including cell proliferation, migration and invasion, cell apoptosis, autophagy, and drug resistance. Until now, the role of m^6^A modification mediated autophagy in cancer drug resistance is still unclear. In this study, we found that m^6^A methyltransferase METTL3-mediated autophagy played an important role in reversing gefitinib resistance by β-elemene in non-small cell lung cancer (NSCLC) cells. Mechanistically, in vitro and in vivo studies indicated that β-elemene could reverse gefitinib resistance in NSCLC cells by inhibiting cell autophagy process in a manner of chloroquine. β-elemene inhibited the autophagy flux by preventing autophagic lysosome acidification, resulting in increasing expression of SQSTM1 and LC3B-II. Moreover, both β-elemene and gefitinib decreased the level of m^6^A methylation of gefitinib resistance cells. METTL3 was higher expressed in lung adenocarcinoma tissues than that of paired normal tissues, and was involved in the gefitinib resistance of NSCLC cells. Furthermore, METTL3 positively regulated autophagy by increasing the critical genes of autophagy pathway such as ATG5 and ATG7. In conclusion, our study unveiled the mechanism of METTL3-mediated autophagy in reversing gefitinib resistance of NSCLC cells by β-elemene, which shed light on providing potential molecular-therapy target and clinical-treatment method in NSCLC patients with gefitinib resistance.

## Introduction

Lung cancer is one of the most common malignant tumors in the world, and its incidence and mortality consistently rank first among malignant tumors^1^. Lung cancer can be divided into two subtypes, non-small cell lung cancer (NSCLC) and small cell lung cancer. NSCLC can be further divided into adenocarcinoma, squamous cell carcinoma, and large cell carcinoma, accounting for about 80% of all lung cancer cases^2,3^. However, the main means of cancer treatment is still surgery combined with radiotherapy and chemotherapy^4^. Despite the increasing use of new anticancer drugs and therapeutic strategies for the treatment of NSCLC, their efficiency is still unsatisfactory^5^. Among them, cancer drug resistance is the main cause of treatment failure and patient death in clinical treatment. Therefore, overcoming the drug resistance of cancer cells has become the key issues to be solved in the field of cancer treatment.

Elemene is an anticancer drug extracted from the Chinese medicine Curcuma Wen yujin, whose main active ingredient is β-elemene. Accumulating evidence suggests that β-elemene has played a huge physiological and pathological role in the treatment of lung cancer, leukemia, liver cancer, cervical cancer, and gastric cancer, though many functional mechanisms of β-elemene have not been discovered^6,7^. β-elemene is used clinically for radiation sensitization and chemotherapy of various tumors, and it can effectively reverse drug resistance^8,9^. Some researchers have shown that β-Ele can reverse the acquired resistance of EGFR inhibitor gefitinib, but its specific mechanism of action is unclear^10,11^. Autophagy is a physiological phenomenon widely existing in eukaryotic cells, which is characterized by transporting abnormal proteins and organelles to lysosomes for degradation^12^. It plays an important role in maintaining cellular metabolism, internal environmental stability, and genomic integrity, whose dysfunction is closely related to the occurrence of various human diseases^13^. Recently, an increasing number of evidence shows that cell autophagy is closely related to drug resistance of cancer cells^14^. High levels of autophagy induced by EGFR-TKIs can protect NSCLC cells from death^15,16^. However, the roles and mechanism of autophagy in reversing gefitinib resistance mediated by β-elemene is still unclear.

M^6^A methylation is a methylation modification found on RNA molecules in the 1970s^17,18^. It mainly regulates the alternative splicing, translation efficiency, and stability of mRNA^19,20^, and thus regulates the expression of target genes. Current research shows that m^6^A methylation is closely related to tumorigenesis and development^21,22^, which provides a new perspective for people to understand tumor cells and guides new directions for the treatment of tumor cells. Therefore, the expression level of m^6^A modification-related genes will be a potential biomarker for molecular diagnosis and prognosis of tumors, and it will also provide new targets for molecular targeted therapy. However, the mechanism of m^6^A methylation modification and gefitinib resistance in NSCLC is unknown.

In this study, we first revealed the role of β-elemene in reversing the resistance of gefitinib in NSCLC. More importantly, we illustrated the molecular mechanism of β-elemene in reversing gefitinib resistance in NSCLC through m^6^A methylation modification mediated autophagy.

## Results

### Construction and characterization analysis of gefitinib-resistant cell lines

**Fig. 1:**
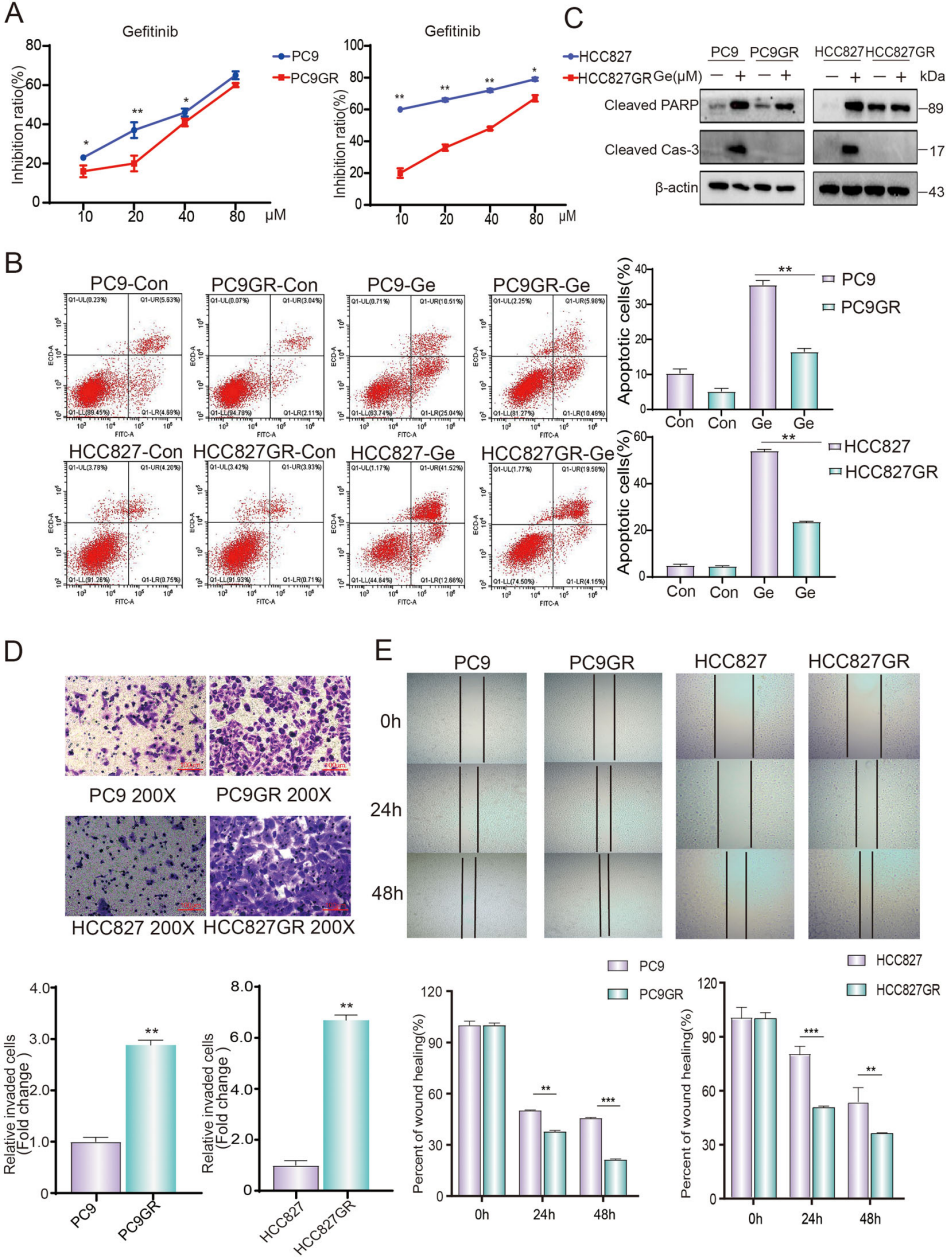
Construction and characterization of gefitinib-resistant cell lines. **A** NSCLC cells were treated with different concentrations of gefitinib for 24 h. The inhibition rate of drugs in NSCLC cells was determined by CCK-8. **B** NSCLC cells were treated with gefitinib for 24 h, stained with Annexin V-FITC and PI, and detected by flow cytometry. **C** Western blotting was performed to study the expression of cleaved caspase-3 and cleaved PARP in NSCLC cells treated with indicated drugs. **D** Representative results of transwell analysis. The pictures were taken 24 h after the boarding (picture display magnification: ×200). **E** Representative results of wound healing assay. Pictures were taken every 24 h at the indicated time (picture display magnification: ×40). Cleaved Cas-3, Cleaved caspase-3; Con, control; Ge, gefitinib. The data were presented as mean ± SD. Asterisk indicates statistically significant difference (***p < 0.001, **p < 0.01, *p < 0.05).

To study the drug resistance of NSCLC cells, NSCLC parental cell lines (PC9 and HCC827) and gefitinib-resistant cell lines (PC9GR and HCC827GR) were incubated at gradient gefitinib concentrations for 24 h. Then, CCK-8 was used to detect the cell viability and IC_50_ value of gefitinib. As expected, the results showed that the cell proliferation activity of the resistant cells was significantly higher than that of parental cells, and the variance was similar between two groups. The IC_50_ value of gefitinib on PC9GR cells was 56.25 μM, which was more than twice as much as PC9 cells (24.1 μM). In another cell pairs, the IC_50_ value of gefitinib on HCC827GR cells was 49.27 μM, which was 11 times more than that of HCC827 cells (4.078 μM) (Fig. 1A). We further determine the apoptosis rate of resistant cells after treating with different concentration gefitinib. The result showed that the apoptosis rates of PC9GR cells and HCC827GR cells treated with 20 μM gefitinib were significantly less than that of their parental cells (Fig. 1B), indicating that resistant cells exhibited much more tolerant than that of parental cells. At the meantime, we have investigated the expression of cleaved caspase-3 and PARP to confirm the cell resistantce against gefitinib. After treating with the same concentration of gefitinib, the expression of cleaved caspase-3 and cleaved PARP in gefitinib-resistant cells was significantly lower than that of parental cells (Fig. 1C). In terms of comparing migration ability of parental and resistant cells, the cell invasion and wound healing assay experiments were performed. The results of transwell and wound healing assay showed that the migration ability of gefitinib-resistant cells was significantly higher than that of parental cells (Fig. 1D, E). All the above results suggested that we successfully constructed gefitinib-resistant cell lines (PC9GR and HCC827GR) which were characterized by higher IC_50_ value of gefitinib, and migration ability.

### β-elemene could reverse gefitinib resistance in NSCLC

**Fig. 2:**
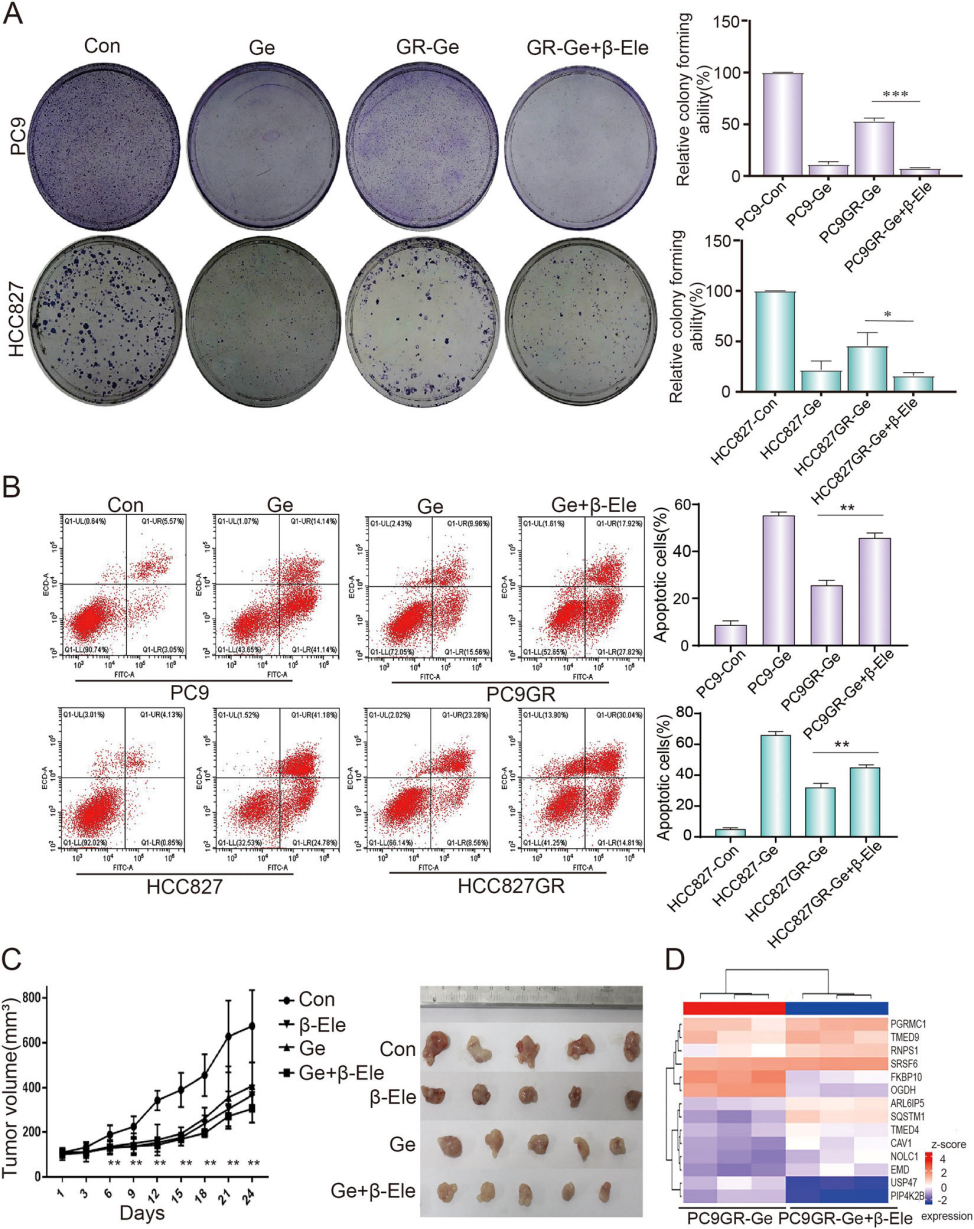
β-elemene could reverse gefitinib resistance in NSCLC. **A** Representative pictures of colony-formation assay. NSCLC cells were treated with indicated concentration of drugs for about two weeks. **B** NSCLC cells were treated with indicated drugs for 24 h, and detected by flow cytometry after Annexin V-FITC/PI dual staining. **C** Effect of indicated drugs on tumor growth in nude mice and quantitative analysis of tumor volume. **D** PC9GR cells were treated with indicated drugs for 24 h. iTARQ sequencing was used to determine the protein expression of treated cells. Con, control; Ge, gefitinib; β-Ele, β-elemene. The data were presented as mean ± SD. Asterisk indicates statistically significant difference (***p < 0.001, **p < 0.01, *p < 0.05).

β-elemene is used clinically for radiation sensitization and chemotherapy of various tumors, and it can effectively reverse drug resistance^8,9^. Therefore, we detected whether β-elemene could reverse gefitinib resistance in NSCLC cells. To determine the inhibitory effect and cytotoxicity of β-elemene in gefitinib-resistant cells under the treatment of gefitinib, we evaluated the synergetic effect of β-elemene and gefitinib. In PC9GR cells, the CCK-8 result showed that the best synergistic effect was obtained when 120 μg/mL β-elemene was combined with 20 μM gefitinib. In HCC827GR cells, the best synergistic effect was obtained when 120 μg/mL β-elemene was combined with 12.5 μM gefitinib (Table S1). The gefitinib-resistant cells were treated with the above drug combination for 24 h in subsequent experiments. The colony-formation assay was performed to investigate the anti-proliferation of β-elemene in combination with gefitinib. After treating with indicated drugs, colony numbers of parental cells were significantly lower than that of resistant cells under the same concentration of gefitinib treatment. Strikingly, colony number of gefitinib treated resistant cells was significantly higher than that of resistant cells exposed to β-elemene in combination with gefitinib (Fig. 2A and Fig. S1). To obtain objective quantification of apoptosis, we used flow cytometry to test the cell apoptosis of resistance cells treated with β-elemene and gefitinib. Annexin V/propidium iodide (AV/PI) dual staining assay suggested that a significant increase number of apoptotic cells was observed in resistant cells in the combinative treatment of β-elemene and gefitinib, comparing with that of gefitinib treated alone (Fig. 2B). These results indicated that β-elemene could successfully reverse drug resistance to gefitinib in NSCLC cells. To study the therapeutic potential of combinative treatment of β-elemene and gefitinib, the animal model of NSCLC PC9GR transplanted in BALB/c nude mice was established to observe anticancer effect of β-elemene and gefitinib. The tumor-bearing mice with similar tumor size were randomly divided into four groups (five mice per group) and intraperitoneally administered 100 μL PBS control, β-elemene (100 mg/kg), gefitinib (100 mg/kg), and β-elemene plus gefitinib, respectively. The growth curve indicated the synergistic effect of anti-tumor activity of β-elemene and gefitinib, comparing with β-elemene or gefitinib treatment alone (Fig. 2C). The tumor harvested from the nude mice was shown in Fig. 2C where the tumor size of drug-treated group was significantly smaller than that of control, especially the group of combinative treatment of β-elemene and gefitinib. Taken together, combinative treatment of β-elemene and gefitinib was sensitive to gefitinb-resistant cells by suppressing cell viability, inducing cell apoptosis, and inhibiting cell proliferation in vitro and in vivo experiments. All these results demonstrated that β-elemene could reverse gefitinib resistance in NSCLC cells. To explore the potential mechanism of β-elemene in reversing gefitinib resistance, iTARQ sequencing was performed to analysis the different protein expression treated with β-elemene and gefitinib. As shown in Fig. 2D, the protein expression of SQSTM1 was significantly increased in combinative treatment of β-elemene and gefitinib, comparing with that of gefitinib treatment, indicating that autophagy might involve in the reversing gefitinib resistance.

### β-elemene reversed gefitinib resistance in NSCLC cells by inhibiting autophagy

**Fig. 3:**
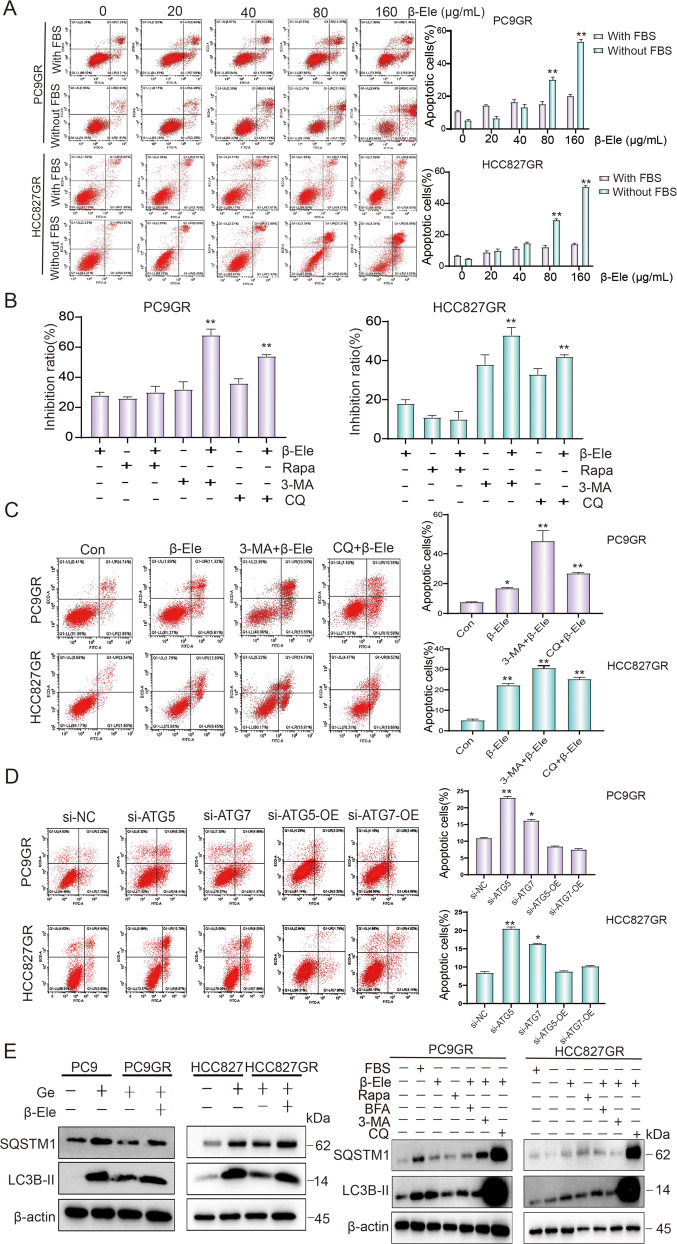
β-elemene could reverse gefitinib resistance by inhibiting autophagy. **A** PC9GR and HCC827GR cells were treated with β-elemene for 24 h in basic and complete medium, the apoptosis rate was measured by flow cytometry after Annexin V-FITC/PI dual staining. **B** Gefitinib-resistant cells were treated with different drugs for 24 h. The inhibition rate of cells was determined by CCK-8. **C** Gefitinib-resistant cells were treated with different drugs for 24 h, the apoptotic cells were measured by flow cytometry after Annexin V-FITC/PI dual staining. **D** The apoptotic cells were measured by flow cytometry after Annexin V-FITC/PI dual staining after changing the expression of ATG5 or ATG7 in gefitinib-resistant cells. **E** Analysis of autophagy-related protein expression of cells threated with various drugs by western blotting. Con, control; Ge, gefitinib; β-Ele, β-elemene; 3-MA, 3-Methyladenine; CQ, chloroquine; Rapa, rapamycin; BFA, brefeldin A; NC, negative control; si, siRNA; OE, overexpression. The data were presented as mean ± SD. Asterisk indicates statistically significant difference (**p < 0.01, *p < 0.05).

Based on existing results and the fact that autophagy plays important role in tumor drug resistance, we speculated that β-elemene could reverse gefitinib resistance in NSCLC cells by autophagy pathway. Since serum-free medium was usually used to induce autophagy, we investigated the sensitivity of β-elemene in serum-containing or serum-free medium by flow cytometry. Under the treatment with no or low concentration β-elemene, there were less apoptotic cells in the serum-free medium than that in serum-containing medium. While the opposite results were observed in treatment with high concentration β-elemene (Fig. 3A). These results suggested that serum-free conditions could induce autophagy to protect cells from apoptosis, and that β-elemene could significantly inhibit this autophagy process. It also indicated that gefitinib-resistant cells are more sensitive to β-elemene in serum-free condition, comparing with that in serum-containing medium. Next, we performed CCK-8 assay to measure the cell viability after autophagy-related drug treatment. As a result, the inhibition ratio of cells was increased after β-elemene treatment, and the inhibition ratio of rapamycin treatment was decreased, while the inhibition ratios of 3-MA and chloroquine treatment were increased, especially increased after combinative treatment of β-elemene and chloroquine or 3-MA (Fig. 3B). To obtain objective quantification of apoptosis, we used flow cytometry to test the cell apoptosis of resistance cells treated with β-elemene and autophagy-related drugs. AV/PI dual staining assay showed that the apoptotic cells was significantly increased after β-elemene treatment. Comparing with treatment of β-elemene alone, the apoptotic cells were significantly increased after combinative treatment of β-elemene and chloroquine or 3-MA (Fig. 3C). The above results suggested that β-elemene, which is similar to autophagy inhibitors, could reduce the viability of gefitinib-resistant cells by inhibiting autophagy. To further check the role of autophagy in gefitinib-resistant cells, we directly knockdown the expression of autophagy-related gene ATG5 or ATG7 to detect apoptotic cells by flow cytometry. As shown in Fig. 3D, downregulation of ATG5 or ATG7 which was confirmed by western blotting (Fig. S2) significantly increased the apoptosis rate of NSCLC gefitinib-resistant cells, compared with that of control siRNA. In addition, rescue of ATG5 or ATG7 which was confirmed by western blotting (Fig. S3) could restore the apoptosis rate of cells to the level of control cells. However, it was still unknown whether β-elemene inhibited phagophore formation at the early stage (like 3-MA) or it inhibited the autophagolysosome formation and acidification at the late stage (like chloroquine). To explore the mechanism of β-elemene in regulating autophagy, western blotting was performed to detect the protein expression levels of gefitinib-resistant cells treated with indicated drugs. As showed in Fig. 3E, similar to the result of chloroquine, the protein expression of LC3B-II and SQSTM1 were significantly increased after β-elemene treatment, especially in combinative treatment of β-elemene and gefitinib. It indicated that β-elemene inhibited cell autophagy in a manner of chloroquine. Therefore, we used chloroquine as a positive control to study the mechanism of β-elemene in the following experiments. Taken together, all these results indicated that β-elemene could reverse gefitinib resistance in NSCLC cells by inhibiting autophagy, and that autophagy plays a critical role in gefitinib resistance by protecting resistant cells from death.

### β-elemene blocked autophagy flux in NSCLC cells

**Fig. 4:**
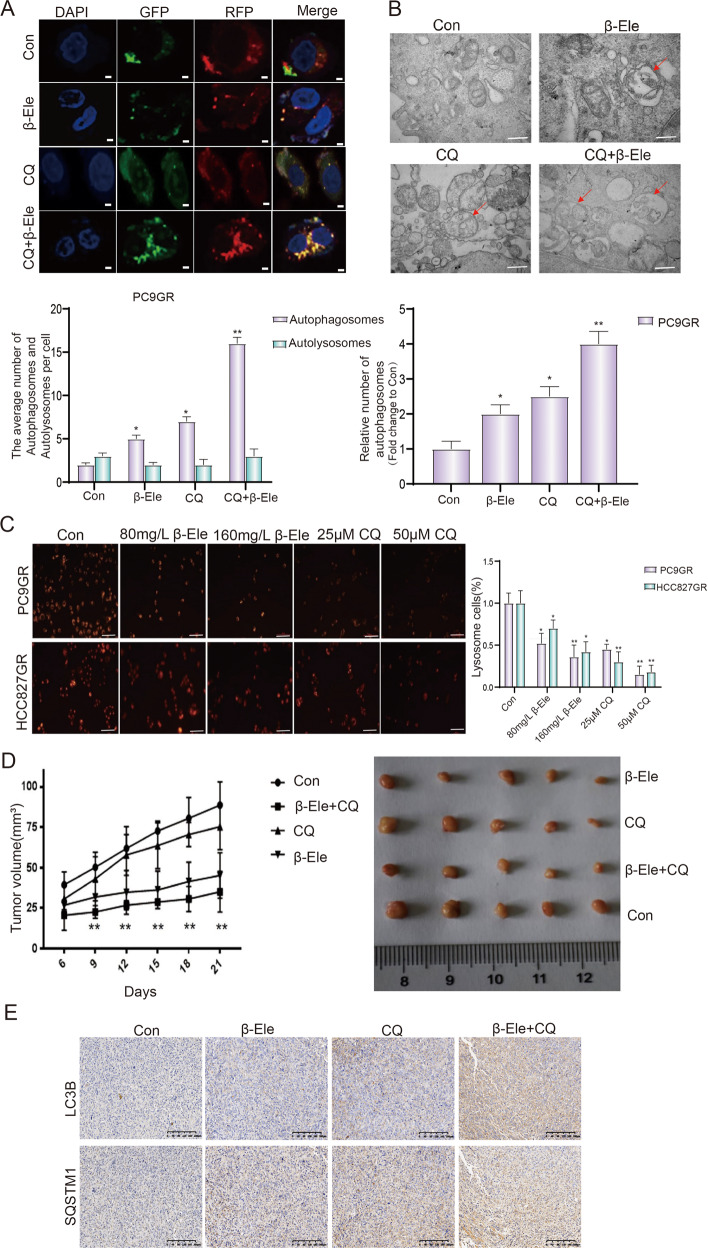
β-elemene blocked autophagy flux in NSCLC cells. **A** NSCLC cells were infected with GFP-RFP-LC3, and then treated with indicated autophagy-related drugs for further 24 h. The representative pictures of fluorescent LC3 spots were shown. Scale bar = 10 μm. **B** PC9GR cells were treated with β-elemene and chloroquine for 24 h, followed by a series of treatments and observed by transmission electron microscopy. Scale bar = 500 nm. **C** PC9GR and HCC827GR cells were treated with β-elemene and chloroquine for 24 h, stained with lyso-tracker red dye, and observed by fluorescence microscopy. Scale bar = 200 μm. **D** Effect of β-elemene and chloroquine on tumor growth in nude mice and quantitative analysis of tumor volume. **E** Immunohistochemical analysis of the expression of LC3B and SQSTM1 in tumor tissues harvested from nude mice with indicated drugs treatment. Con, control; β-Ele, β-elemene; CQ, chloroquine. The data were presented as mean ± SD. Asterisk indicates statistically significant difference (**p < 0.01, *p < 0.05).

The above results indicated that β-elemene inhibited the autophagy in a manner of chloroquine. To further study the mechanism of autophagy regulated by β-elemene, the cells transfected with GFP-RFP-LC3 plasmid was used to investigate the autophagy flow. The results of laser scanning confocal microscope showed that the green particles in the cells treated with chloroquine or β-elemene were significantly increased, especially in the cells of combinative treatment of chloroquine and β-elemene, comparing with those of control. Same tendency was found in the number of yellow particles (Fig. 4A). The green particles are sensitive to the acidic condition in the lysosomal lumen, the red particles indicate the acidic autolysosomes, the yellow particles indicate the colocalization of GFP and RFP fluorescence and represent autophagosomes before fusion with acidic lysosomes^23^. It indicated that β-elemene could increase autophagosomes only, not autolysosomes, which was quite similar to that of the classical autophagy flux blocker chloroquine. To further confirm the role of β-elemene in the process of autophagy, we use the transmission electron microscope to detect the autophagic vacuoles formation after treatment with β-elemene and chloroquine. Much more autophagosomes, characterized by double-membrane compartment and contain electron dense cytoplasmic material and/or organelles without degradation, were found in the cells treated with β-elemene or chloroquine, especially in the cells with combinative treatment of β-elemene and chloroquine (Fig. 4B). These results were consistent with the autophagy flow data, suggesting that the effect of β-elemene in inhibiting autolysosome formation was similar to chloroquine. Chloroquine is known to prevent the maturation of autophagosomes into autolysosomes by raising the lysosomal pH and ultimately inhibit the fusion between autophagosomes and lysosomes^24^. To further prove our speculate that β-elemene could inhibit autophagy by inhibiting the acidification of lysosomes, the lyso-tracker red dye, which is useful to identify acidified vesicular compartments^24^, was used to detect the acidification of lysosomes. As shown in Fig. 4C, the number of red particles, which positively indicated the acidified lysosomes, was decreased with the increasing concentration of β-elemene or chloroquine. These results indicated that β-elemene could inhibit autophagy by impairing lysosomal acidification. Similarly, it has also been reported that gefitinib and AZD9291 can attenuate the acidification of lysosomes, inhibit the fusion of autophagosomes and lysosomes, and eventually inhibits cell autophagy at a late stage^25^. Subsequently, we established a nude mice model with PC9GR cells and evaluated the effects of β-elemene and chloroquine on tumor cell growth in vivo. To simulate the starvation of condition, we feed 80% food of that of normal condition. The tumor-bearing mice with similar tumor size were randomly divided into four groups (five mice per group) and intraperitoneally administered 100 μL PBS control, β-elemene (100 mg/kg), chloroquine (60 mg/kg) and β-elemene plus chloroquine, respectively. The growth curve and harvested tumors showed that average tumor size was significantly reduced after combinative treatment of β-elemene and chloroquine, comparing with that of control or drug treated alone (Fig. 4D). In order to analyze the autophagy flux in tumors treated with indicated drugs, we investigated the expression of LC3B and SQSTM1 by immunohistochemistry. As shown in Fig. 4E, the expression of LC3B and SQSTM1 indicated that the autophagy flux in tumors was consistent with that of NSCLC cells treated with indicated drugs. Taken together, all these results of in vitro and in vivo experiments indicated that β-elemene could reverse gefitinib resistance by inhibiting the autophagy in a similar manner of chloroquine in NSCLC.

### M^6^A methyltransferase METTL3 involved in the regulation of cell autophagy

**Fig. 5:**
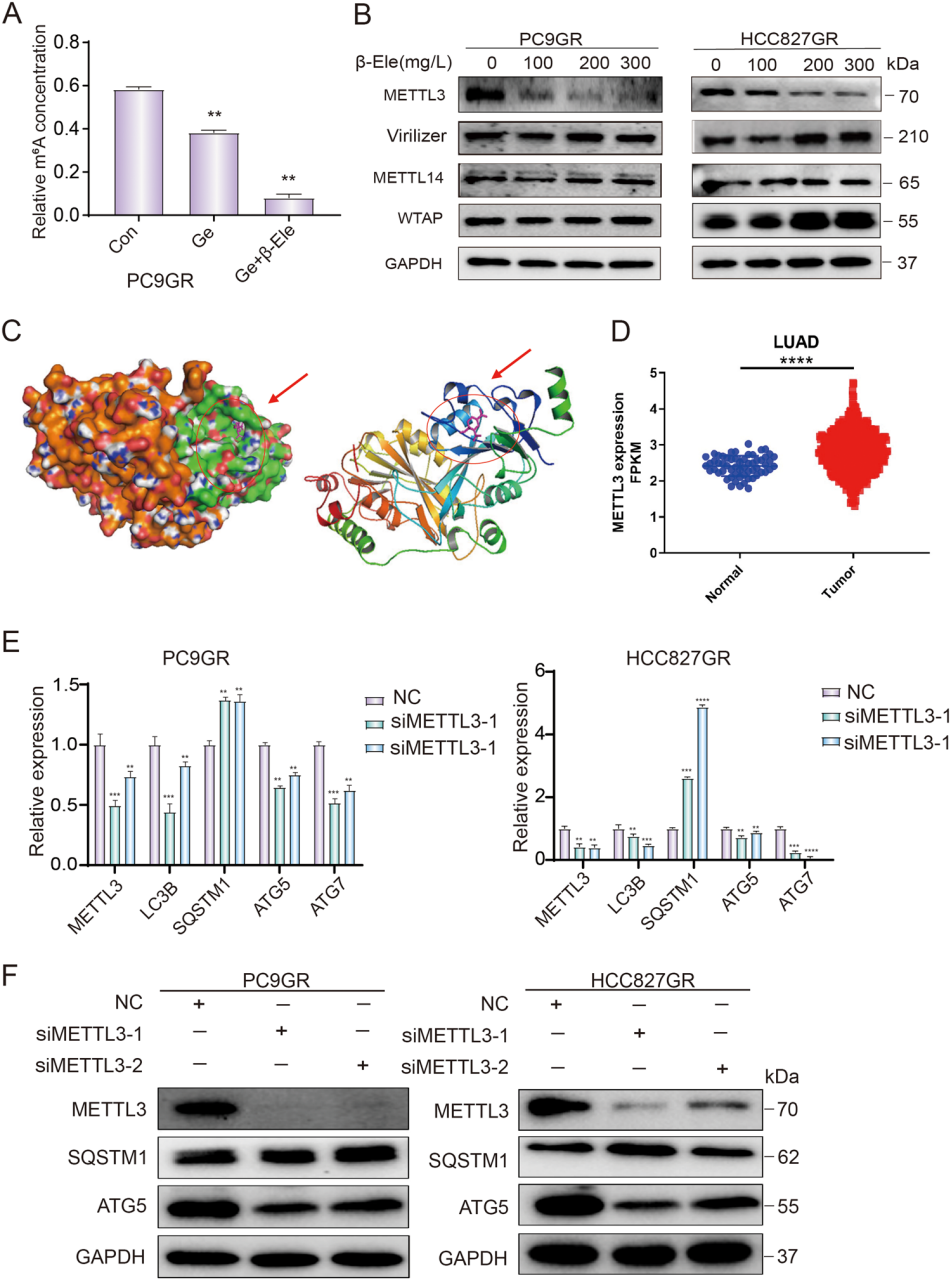
M^6^A methyltransferase METTL3 involved in the regulation of cell autophagy. **A** PC9GR cells were treated with indicated drugs for 24 h, and m^6^A methylation level was detected. **B** Western blotting analysis of m^6^A methyltransferases expression after treatment with different concentrations of β-elemene. **C** Predict the most likely target domain of β-elemene in METTL3 using the online PDB and PubChem database and software of AutoDock Tools. **D** Analysis the expression of METTL3 in cancer tissues and paired normal tissues with TCGA data. **E** qRT-PCR analysis of mRNA expression of autophagy-related genes after downregulation of METTL3. **F** Western blotting analysis of autophagy-related protein expression after downregulation of METTL3. Con, control; Ge, gefitinib; β-Ele, β-elemene; LUAD, lung adenocarcinoma; NC, negative control. The data were presented as mean ± SD. Asterisk indicates statistically significant difference (****p < 0.0001, ***p < 0.001, **p < 0.01).

Most recently, increasing number of articles have reported that m^6^A methylation modification plays a critical role in tumorigenesis and cancer development, including cell autophagy and tumor drug resistance^26^. In order to explore the mechanism of m^6^A methylation modification in reversing gefitinib resistance, we further investigated the effect of β-elemene and gefitinib on m^6^A methylation modification in gefitinib-resistant cells. As shown in Fig. 5A, β-elemene and gefitinib significantly reduced m^6^A methylation levels in NSCLC resistant cells, indicating that m^6^A methylation modification might involve in reversing gefitinib resistance in NSCLC. Since m^6^A methyltransferase is critical and known to install m^6^A to target RNAs, we further investigated whether β-elemene reversing gefitinib resistance by regulating the expression of m^6^A methyltransferases. The result of western blotting showed that β-elemene significantly inhibited the expression of METTL3 in a dose-dependent manner, while it had no obvious effect on the expression of other methyltransferases such as Virilizer, METTL14 and WTAP (Fig. 5B). To further predict the most likely target domain of β-elemene in METTL3, the online PDB and PubChem database and software of AutoDock Tools were used. As shown in Fig. 5C, β-elemene was predicted to directly bind and target on the S-adenosylmethionine binding domain of METTL3. All these results indicated that β-elemene could directly inhibit the activity of METTL3, which needed further investigation. METTL3 is a key component of the large m^6^A methyltransferase complex which is responsible for m^6^A installation and involved in autophagy^26^. To determine the role and mechanism of METTL3 in reversing gefitinib resistance mediated by autophagy, we first analyzed the different protein expression of METTL3 in normal tissues and lung adenocarcinoma tissues by using TCGA database, and found that METTL3 was significantly higher expressed in lung adenocarcinoma patients (Fig. 5D). Subsequently, we detected the potential regulation of METTL3 in autophagy pathway by transfecting siMETTL3 in gefitinib-resistant cells. The results of qRT-PCR experiments showed that knockdown of METTL3 could significantly decrease the mRNA expression of LC3B, ATG5, and ATG7, while increase the mRNA expression of SQSTM1 (Fig. 5E). Similar results were found in the western blotting experiment (Fig. 5F). Altogether, all these results indicated that β-elemene could reverse gefitinib resistance in NSCLC cells by inhibiting METTL3-mediated autophagy. In this autophagy process, METTL3 is positively regulated the autophagy by upregulating the expression of LC3B, ATG5, and ATG7.

## Discussion

Intrinsic or acquired gefitinib resistance is the major obstacle for the successful therapy of NSCLC. It has been reported that β-elemene can reverse the acquired resistance of gefitinib, although its molecular mechanism is unclear^10,11^. In this study, we try to explore the molecular mechanism of β-elemene in reversing gefitinib resistance through regulating cell autophagy. First, in vitro and in vivo results showed that the combination of β-elemene with gefitinib exhibited a better therapeutic effect in the gefitinib-resistant cells by inhibiting the cell viability, colony formation, and inducing cell apoptosis. Further study revealed that β-elemene could increase the autophagosomes only, not autolysosomes, which is characterized by increasing the level of LC3B-II protein and LC3 puncta. Similar results have been found in other research groups. It has been reported that β-elemene can inhibit the activity of the PI3K/Akt/mTOR/p70S6K1 signaling pathway in human NSCLC A549 cells, resulting in increasing the punctate LC3 dots and the levels of LC3-II protein^27,28^. Mechanistically, β-elemene can attenuate the lysosomal acidification and thus impair the fusion between autophagosomes and lysosomes, resulting in preventing the maturation of autophagosomes into autolysosomes, and blocking a late step of autophagy. This mechanism was quite similar to chloroquine which is known as a classical autophagy flux blocker. Meanwhile, much more experiments needed to design to further explore the molecular of β-elemene in impairing the acidification of lysosome, such as its downstream genes which play a critical role in autolysosome formation.

M^6^A methylation is an epigenetic modification on RNA molecules, which mainly affects mRNA stability, nuclear export, and translation initiation through the expression level of mRNA molecules^29^. Accumulating evidence shows that m^6^A methylation plays a critical role in various biological processes, especially in the tumorigenesis and cancer development^30,31^. In our study, we found that both β-elemene and gefitinib could decrease the m^6^A methylation level of gefitinib-resistant cells, indicating the close relationship between m^6^A methylation modification and gefitinib resistance in NSCLC. Further study revealed that β-elemene could inhibit m^6^A methylation by inhibiting the expression of METTL3 rather than other methyltransferases such as Virilizer, METTL14, and WTAP. Further bioinformatics analysis revealed that β-elemene could directly target on the S-adenosylmethionine binding domain of METTL3, and indicated that β-elemene could directly affect the activity of METTL3. Further experiments should be performed in future to explore its molecular mechanism. It has been reported that METTL3 is a key component of the m^6^A methyltransferase complex which is made up of METTL3, METTL14, WTAP and KIAA1429, and plays important roles in autophagy^32^. In this study, METTL3 could positively regulate the autophagy by targeting the autophagy-related genes such as ATG5, ATG7, LC3B, and SQSTM1. However, it still needs further experiments to explore the exact m^6^A methylation mechanism of METTL3 on ATG5, ATG7, LC3B, and SQSTM1. Besides m^6^A methyltransferases, whether there are any other m^6^A modulators (m^6^A demethylase and binding proteins) involved in this autophagy flux regulation is still unknown, which needs further study to explore. Increasing number of evidence shows that m^6^A plays a key role in regulating autophagy^33,34^. For instance, FTO regulates autophagy through targeting ATG5 and ATG7. In detail, knockdown of FTO, a well-known m^6^A demethylase, leads to attenuation of autophagosome formation by decreasing the expression of ATG5 and ATG7^35^. In another study, knockdown of METTL3 can enhance the autophagy flux and inhibit cardiomyocyte apoptosis by m^6^A methylation of TFEB, which is a master regulator of lysosomal biogenesis and autophagy genes^36^.

**Fig. 6:**
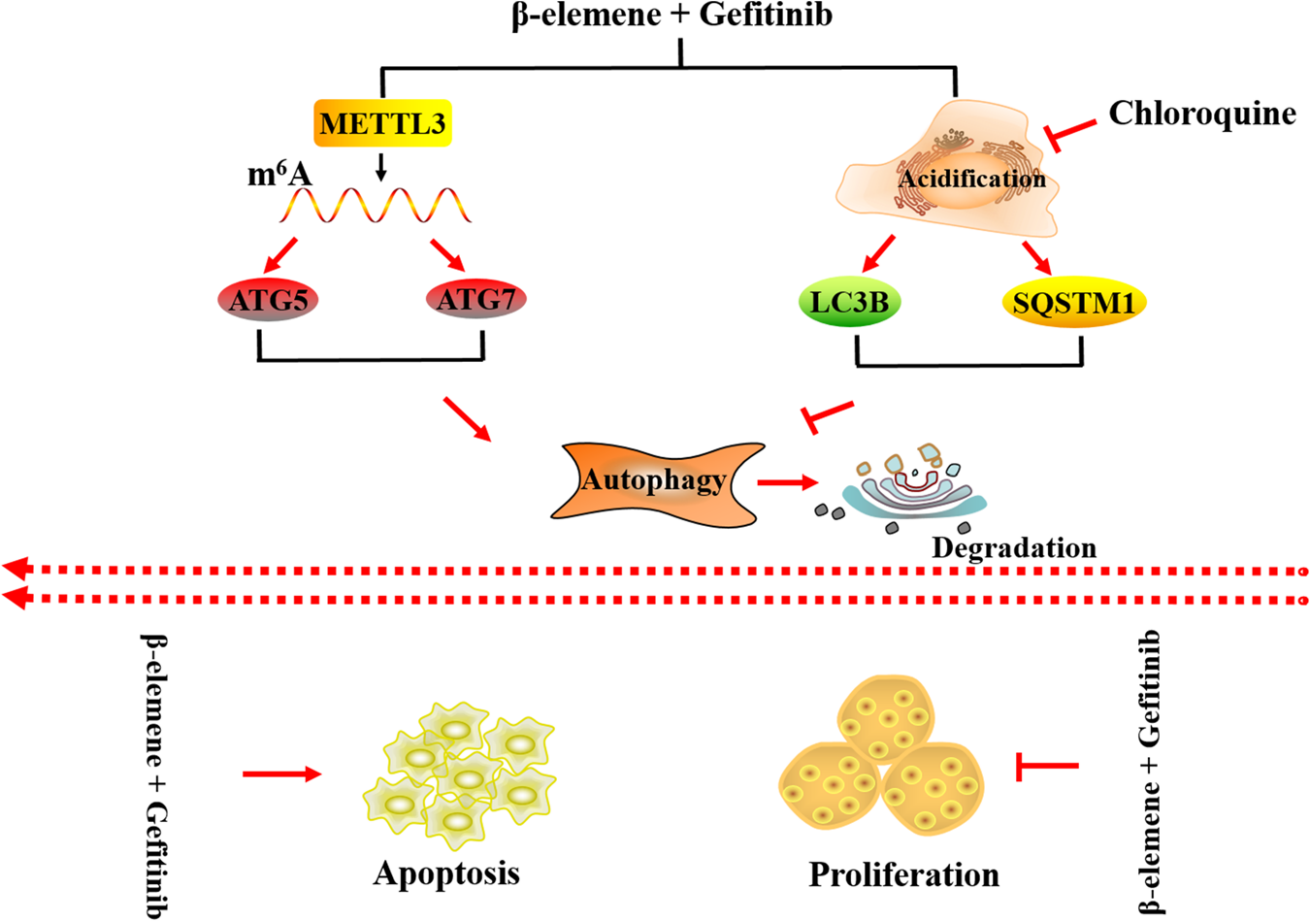
Schematic diagram of the mechanism of β-elemene in reversing gefitinib resistance in NSCLC Cells by m^6^A methyltransferase METTL3 mediated autophagy. β-elemene reverses gefitinib resistance by inducing cell apoptosis, and inhibiting cell proliferation and autophagy. In detail, METTL3 positively regulates autophagy by targeting ATG5 and ATG7, while β-elemene inhibits the autophagy flux by inhibiting acidification of lysosomes in a similar manner to chloroquine, resulting in increasing expression of LC3B and SQSTM1.

In conclusion, we have determined the role of β-elemene in reversing the resistance of gefitinib in NSCLC in vitro and in vivo. Mechanistically, β-elemene can reverse gefitinib resistance by inhibiting the late stage of autophagy in a manner of chloroquine, which inhibits the maturation of autophagosomes into autolysosomes via attenuating the lysosomal acidification. Strikingly, m^6^A methylation modification is involved in this reversing process, and METTL3 can positively regulate this autophagy process by targeting ATG5, ATG7, LC3B, and SQSTM1 (Fig. 6). The result of this study would shed light on providing potential molecular-therapy target and clinical-treatment methods in NSCLC patients with gefitinib resistance.

## Materials and methods

### Cell lines

Human NSCLC cell lines PC9 and HCC827, as parental cells which had no mycoplasma contamination, were derived from the laboratory and identified. To establish a corresponding gefitinib-resistant cell lines PC9GR and HCC827GR, parental cells were treated with a gradient concentration of gefitinib for ~6 months. All cells were cultured with 10% fetal bovine serum and placed in 5% carbon dioxide in air at 37 °C cell incubator.

### Reagents and antibodies

β-elemene (#63965) was purchased from Sigma-Aldrich. Gefitinib (#HY-50895), Rapamycin (#HY-10219), Brefeldin A (#HY-16592), 3-Methyladenine (#HY-19312), and Chloroquine (#HY-17589) were obtained from MedChemExpress. The Cell Counting Kit-8 (#MA0218), Phartmingen annexin V-FITC Apoptosis Detection Kit I (#556547), and Lyso-Tracker Red (#C1046) stain were obtained from Meilunbio, BD Biosciences, and Beyotime, respectively. The PrimeScript RT reagent kit (#RR037A) was purchased from Takara. The antibody against GAPDH (#5174), β-actin (#4970), LC3B (#3868), and SQSTM1 (#16177) were obtained from Cell Signaling Technology. The plasmids for overexpression of ATG5 (#RC210563) and ATG7 (#RC226545) were obtained from ORIGENE. EpiQuik m^6^A RNA Methylation Quantification kit (#710394) was purchased from EPIGENTEK.

### Cell proliferation inhibition assays

The NSCLC cells in logarithmic growth phase were used, 3000 cells per well were put into a 96-well plate, and equal amount of PBS solution was added to the wells around the 96-well plate to prevent edge effects. Then, treated with different concentrations of drug and add 100 μL of the diluted CCK-8 solution to each well according to the instructions and incubate in the incubator for 1–2 h. Finally, the absorption was evaluated by the Multiskan FC microplate reader (Thermo Scientific, USA) and Graphpad 7.0 was used to calculate and analyze the cell proliferation. All experiments were repeated at least three times.

### Cell apoptosis analysis

Apoptotic cells were detected by flow cytometry using Annexin V-FITC/PI dual staining apoptosis detection kit according to the instruction. In short, 3 × 10^5^ cells per well were seeded in 6-well plates and cultured overnight. After treating with indicated concentration of drug, the cells were collected and washes. Added 100 μL 1× binding buffer to resuspend and transfered to a flow tube. Then 5 μL of FITC-Annexin V and 5 μL of PI were added to the sample tube of each sample. The mixed samples were incubated for 15 min at room temperature in the dark. Finally, 300 μL of 1× binding buffer was added to each loading tube, and flow cytometry was performed.

### Cell migration assay

Cultured NSCLC cells were routinely processed. Added 600 μL of 10% FBS containing culture medium to a 24-well plate and placed it in the transwell chamber. Resuspended cell counts in 1% FBS medium and adjusted the cell number to 1 × 10^6^ per mL. Taken 100 μL of the cell suspension into the chamber and incubated in a 37 °C incubator for 24–36 h. Then, the invading cells in the upper chamber were fixed with methanol and stained with 0.25% crystal violet (#C0121, Beyotime, USA). Finally, the chamber was placed upside down with the base film facing up, and taken pictures under an inverted microscope. The experiments were performed in triplicate.

### Wound healing assay

The cells in the logarithmic growth phase were added to a 6-well plate, and the cell number was mastered to be confluent overnight. The next day, the medium was aspirated and scraped to the horizontal line with a yellow pipette, and it was washed twice with fresh medium to remove the scraped cells. The treated cells were placed in 1% fresh medium. Samples were taken at 0, 24, and 48 h to take pictures. Before taking each picture, the culture medium should be replaced with 1% fresh medium. Taking a photo with a microscope (Nikon, Tokyo, Japan) at ×40 magnification to ensure that each photo was taken at the same location. The experiments were performed in triplicate.

### Colony-formation assay

The cells in good logarithmic growth phase were selected for routine processing. Calculated and inoculated 5000 cells into a 10-cm dish. After treating with indicated drug for 10–14 days, the formed cell clones were fixed by adding 2 mL of methanol for 20 min. Subsequently, the methanol was aspirated and a crystal violet solution was added to cover all cell clones at the bottom of the well for 2 h. Finally, photographic recorded the results. The experiments were performed in triplicate.

### Transmission electron microscopy (TEM)

Since electron microscopy is a classical method for observing autophagic compartments, the transmission electron microscopy (TEM) was performed to monitor the process of autolysosome formation after treating with autophagy-related drugs. Briefly, cells treated with autophagy-related reagents were collected after 24 h. The cells were pre-fixed with 2.5% glutaraldehyde at room temperature, and fixed with 1% osmium tetroxide for 2 h. Subsequently, the samples were gradually dehydrated with increasing concentrations of ethanol and acetone, and embedded in araldite. Finally, a 50–60 nm section was prepared on an LKB-1 ultrathin microtome and transferred to a copper mesh, and it was photographed with a JEM-1400 plus transmission electron microscope (JEOL, Japan).

### Measurement of autophagic flux

To measure autophagic flux, NSCLC cells were transiently transfected with the GFP-RFP-LC3 plasmid using Lipofectamine 2000 according to manufacturer instructions. After treating with autophagy-related drugs, taken fluorescent photos with confocal laser scanning microscope (#FV3000RS, Olympus, Japan). Autophagic flux was determined by the presence of yellow puncta and red puncta. Among them, the yellow spot is the overlap of the red and green fluorescent signals.

### Lyso-tracker red staining

NSCLC resistant cells in logarithmic growth phase were selected for routine collection. In total, 3 × 10^5^ cells were seeded per well and cultured in 6-well plates overnight. After treating with indicated concentrations of β-elemene and chloroquine, added a certain proportion of lyso-tracker red probe into the cells, and incubated for 15 min at room temperature according to the instructions. Finally, taken pictures with fluorescence microscope (#Eclipse Ci-L, Nikon, Japan).

### Western blotting

The cells were directly scraped off with a cell scraper to centrifuge, then the cells were washed twice with PBS and lysed with RIPA buffer. After measurement of the protein concentration, the same amount of total protein was used for SDS-PAGE. The isolated proteins were transferred to a PVDF membrane at 250 mA for 90 min. Subsequently, the membrane was blocked with 5% skim milk for 1 h at room temperature and incubated overnight at 4 °C with primary antibody (1:1000). Afterward, washed and incubated with HRP-conjugated secondary antibody (1:1000) for 1 h. Finally, the detection was performed using an ECL kit (#1705061, Bio-Rad, USA) and visualized with ChemiDoc Imaging System (Bio-Rad, USA).

### Quantitative real-time PCR

The total RNA was isolated from NSCLC cell samples using Trizol reagent and adjusted to 200 μg/mL. 0.5 μg total RNA was used for reverse transcription. Finally, the designed primers (Table S2) were used for quantitative real-time PCR in Bio-Rad PCR instrument, and each sample was analyzed in triplicate.

### iTARQ sequencing analysis

After treating with indicated drugs for 24 h, the NSCLC gefitinib-resistant cells were collected and sent to Zhejiang Quanchuan Biological Company (China) for iTARQ sequencing. Each sample was prepared and analyzed in triplicate.

### In vivo subcutaneous tumor model

All in vivo experiments were approved by the Animal Protection Committee of Hangzhou Normal University. NSCLC gefitinib-resistant cells (5 × 10^6^ cells in 100 μL PBS) were injected subcutaneously into the lateral surface of the left abdomen of 6-week-old female BALB/c nude mice (at least five mice per group to ensure accuracy). All the mice with tumor were randomly grouped and intraperitoneal administration with indicated drugs or PBS control for indicated days. Tumor volume was assessed by investigators who were blinded to the group allocation every 2 days for about 1 month. Tumor volume was calculated by the following formula: (short diameter)^2^ × (long diameter)/2.

### Statistical analysis

All graphics in this paper were statistically analyzed by GraphPad Prism 7.0, and SD detection was performed with the results of three experiments. Among them, *p* < 0.05 and *p* < 0.01 were considered statistically significant and obviously statistically significant. Finally, all graphics were organized in AI drawing tools.

## Supplementary information

Supplementary Table S1 and S2

Supplementary Figure Legends

Supplemental Figure S1

Supplemental Figure S2

Supplemental Figure S3
