# Supplementary material for: The mechanism of m6A methyltransferase METTL3-mediated autophagy in reversing gefitinib resistance in NSCLC cells by β-elemene
Source: Cell Death Dis. 2020 Nov 11;11(11):969. doi: 10.1038/s41419-020-03148-8 (PMC7658972; doi:10.1038/s41419-020-03148-8)
Supplement: Supplementary file 1 — Supplementary Table S1 and S2 [file 41419_2020_3148_MOESM1_ESM.docx]

**Supplementary tables**

**Table S1.**

| **PC9GR** | | |  | **HCC827GR** | | |
| --- | --- | --- | --- | --- | --- | --- |
| **Gefitinib (μM)** | **β-elemene (μg/mL)** | **CI** |  | **Gefitinib (μM)** | **β-elemene (μg/mL)** | **CI** |
| 20 | 150 | 0.845 |  | 12.5 | 150 | 0.817 |
| 20 | 120 | 0.632 |  | 12.5 | 120 | 0.813 |
| 20 | 100 | 0.820 |  | 12.5 | 100 | 0.857 |
| 20 | 80 | 0.724 |  | 12.5 | 80 | 0.946 |
| 20 | 50 | 0.771 |  | 12.5 | 50 | 1.032 |
| 10 | 150 | 0.905 |  | 6.25 | 150 | 2.012 |
| 10 | 120 | 0.928 |  | 6.25 | 120 | 1.470 |
| 10 | 100 | 0.726 |  | 6.25 | 100 | 1.165 |
| 10 | 80 | 0.765 |  | 6.25 | 80 | 0.973 |
| 10 | 50 | 0.687 |  | 6.25 | 50 | 0.870 |

**Table S2.**

| Primer name | Primer sequence |
| --- | --- |
| GAPDHF | 5′-GCGGAGAGCTTTAAAGTGCG-3′ |
| GAPDHR | 5′-GCGGAGAGCTTTAAAGTGCG-3′ |
| METTL3F | 5′-GAGGAGTGCATGAAAGCCAG-3′ |
| METTL3R | 5′-GGCCTCAGAATCCATGCAAG-3′ |
| LC3BF | 5′-AAGGCGCTTACAGCTCAATG-3′ |
| LC3BR | 5′-CTGGGAGGCATAGACCATGT-3′ |
| SQSTM1F | 5′-GGCGACCAGTTCTCCTGAATC- 3′ |
| SQSTM1R | 5′-GGCAGCATGAAAGTTAGCAGA- 3′ |
| ATG5F | 5′-AAAGATGTGCTTCGAGATGTGT-3′ |
| ATG5R | 5′-CACTTTGTCAGTTACCAACGTCA-3′ |
| ATG7F | 5′-CTGCCAGCTCGCTTAACATTG-3′ |
| ATG7R | 5′-CTTGTTGAGGAGTACAGGGTTTT-3′ |
