# Supplementary material for: The mechanism of m6A methyltransferase METTL3-mediated autophagy in reversing gefitinib resistance in NSCLC cells by β-elemene
Source: Cell Death Dis. 2020 Nov 11;11(11):969. doi: 10.1038/s41419-020-03148-8 (PMC7658972; doi:10.1038/s41419-020-03148-8)
Supplement: Supplementary file 2 — Supplementary Figure Legends [file 41419_2020_3148_MOESM2_ESM.docx]

**Supplementary figure and table legends**

**Figure S1** The representative pictures of β-elemene in reversing gefitinib resistance in NSCLC cells treated with β-elemene and gefitinib. Con, control; Ge, gefitinib; β-Ele, β-elemene; GR, gefitinib resistant cells. Scale bar =100 μm.

**Figure S2** Western blotting analysis of the expression of ATG5 (**a**/**c**) or ATG7 (**b**/**d**) in NSCLC gefitinib resistant cells after knockdown of indicated genes, the relative ratio of knockdown for ATG5 or ATG7 was analyzed by Image J. NC, negative control; si, siRNA.

**Figure S3** Western blotting analysis of the expression of ATG5 or ATG7 in NSCLC gefitinib resistant cells after indicated treatment. NC, negative control; si, siRNA; OE, over expression.

**Table S1.** The CI of β-elemene and gefitinib in gefitinib-resistant cells according to the method of Soriano. Among them, 0.9 ≤ CI <1.1 indicates a superimposed effect, 0.8 ≤ CI <0.9 indicates a low-level synergy, 0.6 ≤ CI <0.8 indicates a moderate synergy, and 0.4 ≤ CI <0.6 indicates a high synergy, 0.2≤CI <0.4 indicates a strong synergy.

**Table S2.** Primer sequence listed as follows. Primers were designed using Primer 5 online database and synthesized by Qingke Bioprimer company.
