# Supplementary figures and images for: The mechanism of m6A methyltransferase METTL3-mediated autophagy in reversing gefitinib resistance in NSCLC cells by β-elemene
Source: Cell Death Dis. 2020 Nov 11;11(11):969. doi: 10.1038/s41419-020-03148-8 (PMC7658972; doi:10.1038/s41419-020-03148-8)

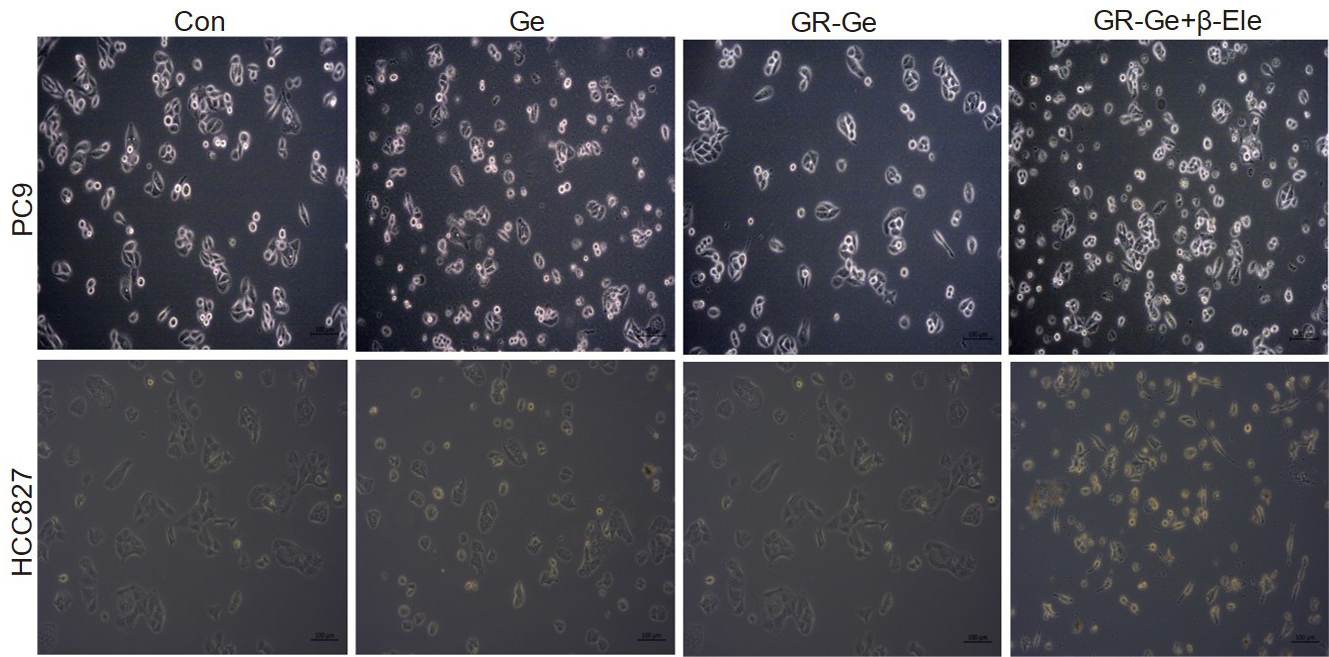

Supplement: Supplementary file 3 — Supplemental Figure S1 [file 41419_2020_3148_MOESM3_ESM.tif]

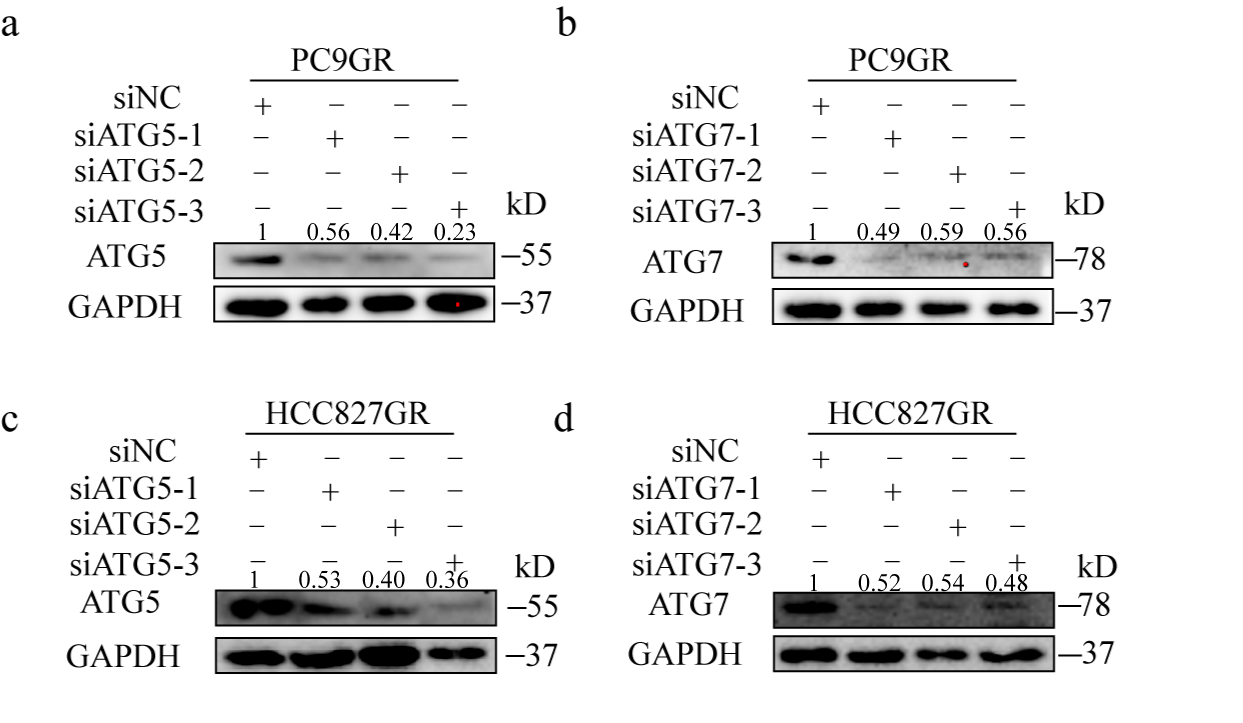

Supplement: Supplementary file 4 — Supplemental Figure S2 [file 41419_2020_3148_MOESM4_ESM.tif]

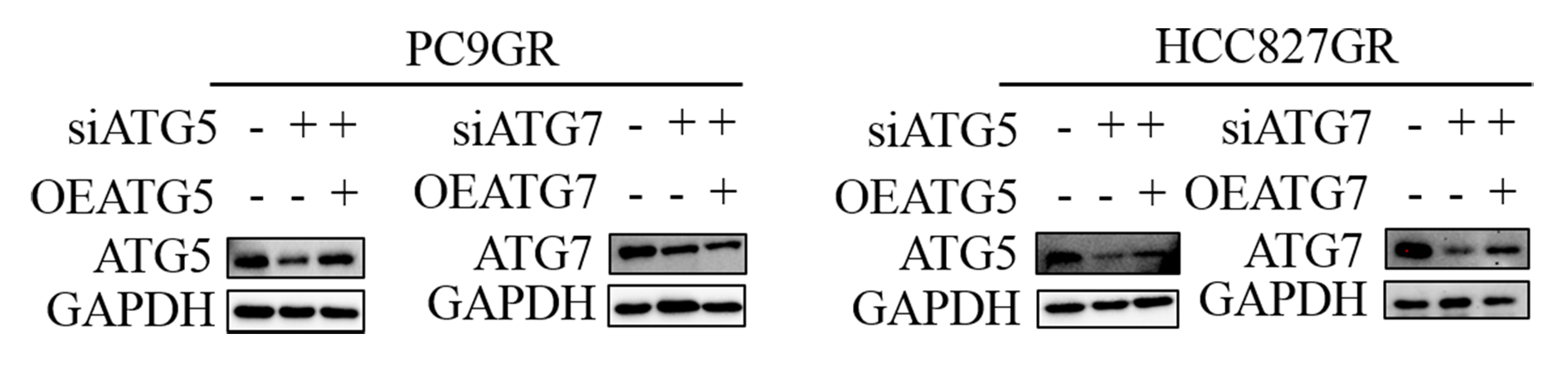

Supplement: Supplementary file 5 — Supplemental Figure S3 [file 41419_2020_3148_MOESM5_ESM.tif]
